# Induction of Pyroptosis: A Promising Strategy for Cancer Treatment

**DOI:** 10.3389/fonc.2021.635774

**Published:** 2021-02-26

**Authors:** Lei Wang, Xiaowei Qin, Jianmin Liang, Pengfei Ge

**Affiliations:** ^1^ Department of Neurosurgery, First Hospital of Jilin University, Jilin University, Changchun, China; ^2^ Department of Pediatric Neurology, First Hospital of Jilin University, Jilin University, Changchun, China

**Keywords:** pyroptosis, cancer, inflammasome, antitumor immunity, gasdermin, caspase

## Abstract

Pyroptosis, a lytic pro-inflammatory type of programmed cell death, has been widely studied in diverse inflammatory disease models. Membrane perforation and cell swelling induced by cleaved gasdermin family members is the main characteristic of pyroptosis. Emerging evidence has revealed a complicated relationship between pyroptosis and cancer. On the one hand, as inflammatory cell death, pyroptosis provides a comfortable environment for tumor proliferation. On the other hand, excessive activation of pyroptosis can inhibit the development of tumor cells. In this review, we first summarized the latest progress about the molecular mechanism of pyroptosis. Then, members from gasdermin family, the central molecules of pyroptosis which formed pores on the cell membrane, were highlighted. In the second part of this review, we summarized drugs that induced pyroptosis in different tumors and their concrete mechanisms based on recent literature reports. In the final section, we discussed several hotspots in pyroptosis and cancer therapy, which will point out the direction of sequent research. In brief, inducing pyroptosis in cancer cells is a promising strategy for cancer therapy.

## Introduction

The dynamic balance among cell proliferation, differentiation and death plays an important role in the physiological and pathological processes of multicellular organisms (1). Based on considerable research on cell death mechanisms, biologists have found multiple cell death forms such as apoptosis, necrosis, necroptosis, ferroptosis, etc (2). In 1999, researchers found that caspase-1 was activated in the cell death induced by Salmonella. The cell death induced by Salmonella was initially considered as caspase-1-dependent apoptosis (3). However, in Brennan and Cookson’s research, Salmonella-induced cell death exhibited utterly different characteristics from apoptosis. Caspase-3 and PARP1 were not activated in Salmonella-infected macrophages. What’s more, the cell membrane integrity of infected macrophages was destroyed while apoptosis cells usually had intact membranes (4). All of the differences above indicated that Salmonella-induced macrophage death was distinct from the well-known apoptosis. Thus, pyroptosis, an inflammatory programmed cell death (PCD), was proposed to elucidate such a cell death phenomenon in 2001 (5). The main characteristics of pyroptosis include membrane perforation, cell swelling, the release of cellular content, chromatin condensation and DNA fragmentation (6, 7). In the canonical inflammasome pathway of pyroptosis, activated caspase-1 promotes the maturation of IL-1β and IL-18. Meanwhile, activated caspase-1 also cleaves GSDMD into the C-terminal domain and N-terminal domain. Then the GSDMD-NT forms pores in the plasma membrane (6, 8, 9). In vertebrates, pyroptosis is beneficial for the clearance of pathogen and enhancing innate immunity (10). As a promising research direction, pyroptosis plays important roles in diverse diseases such as hepatitis, atherosclerosis, neurodegeneration, tumor, etc. (1, 11–13). Pyroptosis performs dual effects on tumors. On the one hand, pyroptosis-associated inflammatory cytokines and pathways promote tumor growth, invasion and drug resistance (14, 15). On the other hand, inducing pyroptosis directly suppresses tumor proliferation (16). Therefore, relevant studies on pyroptosis have positive significance for developing new tumor treatment regimens, reducing chemotherapeutic drug resistance and improving the life quality of tumor patients. In this review, we aimed to summarize and evaluate the recent studies focused on pyroptosis and cancer therapy. Subsequently, several hotspots in this field were discussed in detail. The review of earlier work could help us to define the direction of future research. We hope there will be more high-quality studies of tumor therapy based on pyroptosis in the future.

## Signal Pathways of Pyroptosis

### Canonical Inflammasome Pathway

Activation of the inflammasome is the basis for caspase-1-dependent pyroptosis. The inflammasome complex is usually composed of pattern recognition receptors (PRRs), inflammatory caspases and in some cases, an adapter protein that connects these proteins (17). The most common PRRs in the inflammasome complex comprise NLRP1, NLRP3, NLRC4, and AIM2 (18), which could be activated by various stimulation. For example, NLRP1 identifies the Bacillus anthracis lethal toxin to induce pyroptosis (19). NLRC4 is responded to bacterial protein, while AIM2 is mainly activated by bacteria or double-stranded DNA in cells infected by viruses. NLRP3, the best-studied PRRs, can be activated by various factors, including bacterial toxins, viral double-stranded RNA, adenosine triphosphoric acid, ROS and endogenous damage signals (10). Activated NLRP3 or AIM2 forms a macromolecule complex with adapter protein ASC and pro-caspase-1, further initiating pyroptosis. Besides, receptor proteins with caspase recruitment domain (CARD) such as NLRP1 or NLRC4 also recruit pro-caspase-1 and promote its proteolysis directly (15). Earlier research indicated that caspase-1 provoked the maturation of inflammatory cytokines such as IL-1β, IL-18, although the specific mechanism of caspase-1 inducing pyroptosis was elusive. In 2015, Feng Shao and colleagues found GSDMD knockdown in iBMDM cells reversed caspase-1-dependent pyroptosis, which demonstrated the crucial role of GSDMD in pyroptosis. GSDMD, a member of gasdermin family, is highly conserved among mammalians. During the occurrence of pyroptosis, caspase-1 cleaves GSDMD into GSDMD-CT and GSDMD-NT with molecular weights of 22kd and 31kd respectively. Subsequently, due to their lipophilic property, GSDMD-NT is aggregated in the inner side of the cytomembrane to generate lots of pores, which leads to cell swelling, membrane perforation and release of cellular content such as IL-1β and IL-18. Binding GSDMD-CT to GSDMD-NT inhibits all changes above (8, 9, 20).

### Noncanonical Inflammasome Pathway

The noncanonical pyroptosis pathway is independent of the inflammasome complex. Relevant studies have shown that bacterial Lipopolysaccharide (LPS) directly activated human caspase-4/5 and mouse caspase-11. Then the activated caspases cleaved GSDMD to perforate cell membrane (21, 22). In addition, although caspase-4/5/11 doesn’t directly trigger hydrolysis of IL-1β and IL-18, the potassium efflux from ruptured cells still induces NLRP3/caspase-1 activation, ultimately leading to maturation and release of IL-1β and IL-18. The maturation of IL-1β and IL-18 in the noncanonical pathway is independent of caspase-4/5/11 (20, 23, 24).

### Other Pathways Inducing Pyroptosis

**Figure 1:**
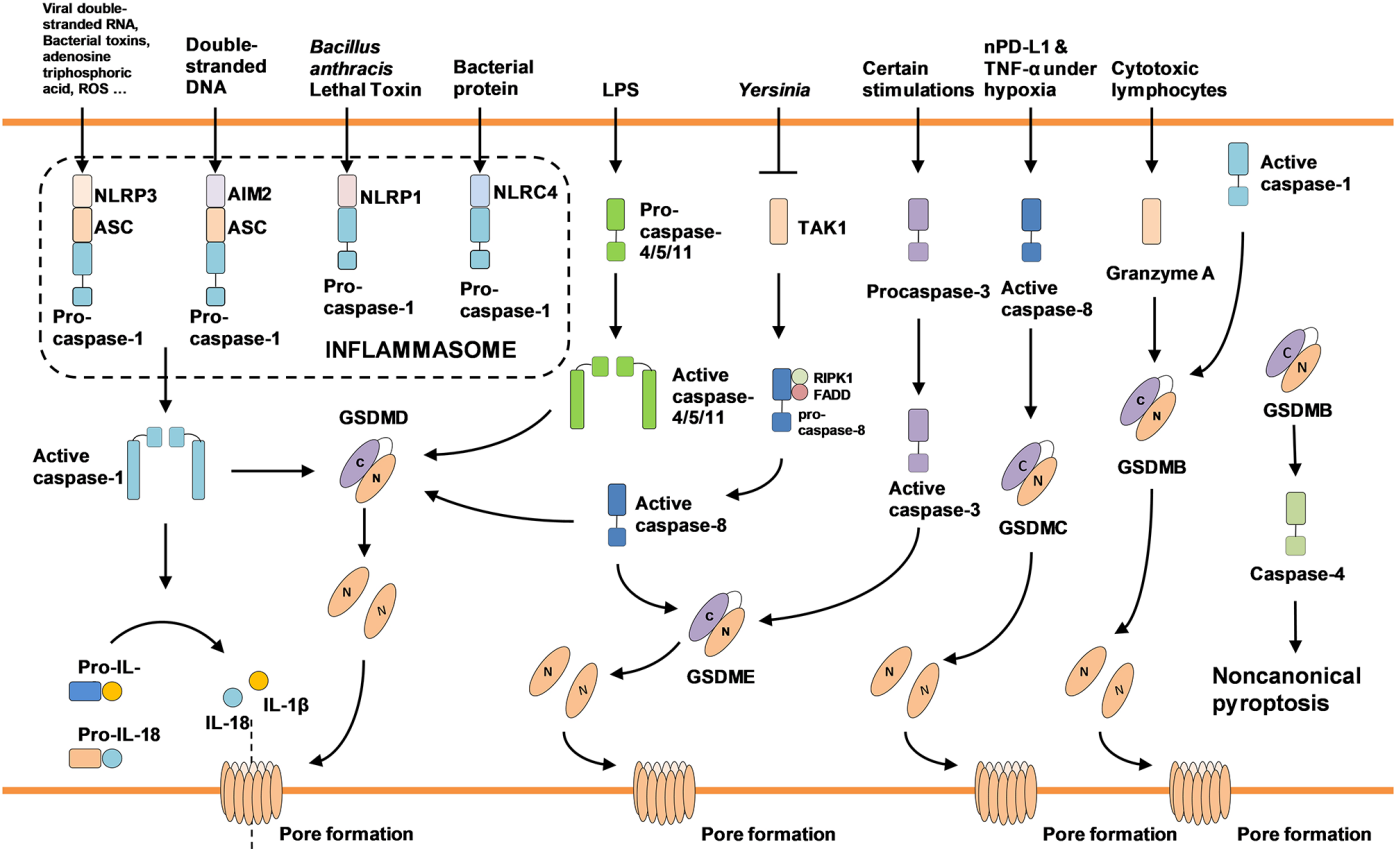
Pyroptosis-associated molecules and signaling pathways.

Apart from caspase-1/4/5/11, some non-inflammatory caspases also cause pyroptosis. GSDMD and GSDME, both of which belong to the gasdermin family, could be cleaved to generate an N-terminal with a perforating effect. The morphology of TNF-α-induced Hela cell death was similar to pyroptosis accompanied by activation of caspase-3/7 and GSDME. Knocking down caspase-3 switched pyroptosis to apoptosis through inactivating GSDME. Thus, researchers hypothesized that activated caspase-3, induced by TNF-α, independently cleaved GSDME to promote pyroptosis. Subsequent studies confirmed that caspase-3 triggered GSDME-dependent pyroptosis in high GSDME expression cells in contrast to apoptosis in cells with low GSDME expression (25–27). Another non-inflammatory caspase inducing pyroptosis is caspase-8. *Yersinia* activates RIPK1/caspase-8 pathway by suppressing TGF-β activated kinase-1 (TAK1). After that, activated caspase-8 cleaves GSDMD and GSDME to elicit pyroptosis (28–30). Besides, caspase-6 was recently found to enhance the interaction between RIPK3 and ZBP1 to activate NLRP3/caspase-1 signal pathway (31). As a newfound cell death pathway, the molecular mechanisms of pyroptosis deserve further investigation (**Figure 1**).

## Gasdermin Family Plays a Central Role in Pyroptosis

So far, researchers have classified the human gasdermin family into six categories according to their distinction of conserved sequence, including GSDMA, GSDMB, GSDMC, GSDMD, GSDME/DFNA5, and DFNB59 (32). Except for DFNB59, the rest molecules from gasdermin family are activated by cleaving into C-terminal and N-terminal. Owing to the lipophilicity and perforating ability of the N-terminal, the whole gasdermin family cause pyroptosis, which is inhibited by binding to the C-terminal (9). Early studies on pyroptosis mainly focused on inflammatory caspase-1/4/5/11 and GSDMD. Thus, pyroptosis was defined as inflammatory cell death. With in-depth research, scientists turned their attention to gasdermin family that directly destroyed cell membranes. Due to the pore formation property of gasdermin family, Some researchers redefined pyroptosis as gasdermin-mediated programmed cell death (20). Whether participation of caspase should be necessary for pyroptosis is still controversial.

GSDMD is the earliest discovered pyroptosis-associated gasdermin. In 2015, two separate teams led by Feng Shao and Vishva M Dixit demonstrated respectively that GSDMD was involved in canonical and noncanonical pyroptosis (8, 22). Up to now, it has been found that GSDMD was cleaved by inflammatory caspase-1/4/5/11 or non-inflammatory caspase-8 to elicit PCD. In neutrophils, GSDMD is cleaved by neutrophil elastase at C268 (seven amino acids upstream of the caspase cleavage site) to induce pyroptosis (33). GSDME is mainly activated by non-inflammatory caspase (caspase-3/8). Mutations in mice GSDMA3 exhibit hair-loss phenotypes (12). Specific GSDMA3 mutations (T278P, L343P, Y344C, A348T, and 412EA) were found to interfere with the combination of GSDMA3-CT and GSDMA3-NT. Therefore, researchers speculated that the hair-loss was triggered by GSDMA3-NT-induced pyroptosis (8). Different from Feng Shao’s view that caspase-1 was unable to activate GSDMB, Panganiban found GSDMB was cleaved at the D236 position to induce pyroptosis (34). Another recent study demonstrated that GSDMB was cleaved by granzyme A released from cytotoxic lymphocytes, promoting our comprehension of cytotoxicity of lymphocytes (35). GSDMB is upregulated in patients with sepsis or Crohn’s disease. Full-length GSDMB binds to caspase-4 and activates noncanonical pyroptosis through caspase-4/GSDMD pathway. High expression of GSDMB is not directly involved in membrane perforating (36). Similar to other gasdermins, the N-terminal of GSDMC can lead to pore formation (9). In recent research, GSDMC expression was transcriptionally activated by nPD-L1 under hypoxia. The overexpressed GSDMC was subsequently cleaved by caspase-8 and switched TNF-α induced apoptosis to pyroptosis (37). Although extensive work has been finished in this field, it remains to be further explored whether there are undiscovered mechanisms for gasdermins to induce pyroptosis.

## Similarities, Differences, and Cross-Talk Among Pyroptosis, Apoptosis, and Necroptosis

Pyroptosis, apoptosis and necroptosis are three PCD pathways that have been intensively investigated recently. Although induced by distinct mechanisms, the three modes of cell death share many similarities and could be activated alone or simultaneously depending on diverse cellular contexts (26, 30, 38, 39). To better comprehend PCD in cancer treatment, it is necessary to distinguish their main characteristics briefly (**Table 1**). Apoptosis is one of the earliest defined PCD that widely participates in many biological processes such as embryogenesis, aging, infection and tumor regression. The major morphological characteristics of apoptosis including membrane blebbing, cell shrinkage, chromatin condensation, nuclear fragmentation, and the formation of apoptotic bodies (40). Distinct from apoptotic cells that retain intact membrane, cell membrane under pyroptosis and necroptosis are perforated by gasdermins and MLKL. Therefore, cell swelling and membrane rupture are usually observed in pyroptotic and necroptotic cells (6, 41, 42). Since the perforation triggered by GSDMD is less ion-selective than MLKL, the cell swelling is milder in pyroptosis (43, 44). Consistent with apoptosis, DNA fragmentation and chromatin condensation are displayed in pyroptotic cells. However, pyroptosis exhibits DNA damage at a lower intensity with an integral nucleus (6). Besides, intact nuclei are also observed in necroptotic cells (42). As a result of cell membrane damage, several damage-associated molecular patterns (DAMPs) and inflammatory factors are released from pyroptotic and necroptotic cells, which subsequently triggered a potent inflammatory response (45). Reversely, apoptosis is traditionally considered as a non-inflammatory process although the emission of particular DAMPs such as ATP and HMGB1 were demonstrated in apoptosis according to recent literature (46, 47). In terms of molecular mechanisms, caspases are widely involved in the three PCDs. In canonical and noncanonical pyroptosis, inflammatory caspases (e.g., caspase-1, -4, -5, -11) are generally activated to initiate the cleavage of gasdermins (18). Another group of caspases (e.g., caspase-2, -3, -6, -7, -8, -9, -10) are thought to participate in the initiation and execution of apoptotic pathways (48). Although caspases are dispensable for necroptosis, the inactivation or absence of caspase-8 is a prerequisite for necrosome formation and necroptosis activation (45). Though pyroptosis, apoptosis and necroptosis are three distinct PCDs with various molecular mechanisms and morphological characteristics, recent reports indicated the existence of crosstalk among them. Caspase-3/8, general associated with apoptosis, were demonstrated to cleave GSDME or GSDMD and induce pyroptosis (25, 29). In contrast, the inflammatory caspase-1 could activate apoptosis in the absence of GSDMD (49). Crosstalk between pyroptosis and necroptosis was also discovered in recent years. MLKL, the executioner of necroptosis, activates NLRP3 inflammasome to promote the maturation of IL-1β and IL-18 (50). However, GSDMD is dispensable for the release of cytokines from necroptotic cells (51).

**Table 1:** Similarities and differences among pyroptosis, apoptosis, and necroptosis.

Items	Pyroptosis	Apoptosis	Necroptosis
Morphology of cell membrane	Membrane permeabilization, cell swelling	Membrane blebbing, cell shrinkage	Membrane permeabilization, cell swelling
Morphology of nucleus	Integral nucleus, chromatin condensation	Nuclear fragmentation, chromatin condensation	Integral nucleus
Special constructions	Pyroptotic bodies	Apoptotic bodies	None
Release of cellular contents	DAMPs, inflammatory cytokines	Particular DAMPs such as ATP, HMGB1	DAMPs, inflammatory cytokines
Key molecules	Caspase-1/4/5/11, GSDMD, GSDME	Caspase-2/3/6/7/8/9/10	RIP1, RIP3, MLKL

## Application of Pyroptosis in Tumor Treatment

As inflammatory cell death, the role of pyroptosis in tumor progression and suppression remains elusive. The chronic inflammation caused by inflammatory mediators released from pyroptotic tissues increases the risk of tumorigenesis. HMGB1 released from pyroptotic epithelial cells promotes tumorigenesis of colitis-associated colorectal cancer by activating ERK 1/2 pathway (52). A recent study showed that pyroptosis triggered in the central hypoxic region of tumor provoked tumor progression and correlated with reduced survival (37). However, more researchers have found that some anti-cancer agents can induce pyroptosis to suppress cancers’ occurrence and development (25). One possible explanation for the dual effects of pyroptosis is that the chronic inflammation activation facilitates tumor progression, while acute activation of pyroptosis leads to necrotic cell death and represses tumor development (37). As nonapoptotic cell death, pyroptosis can overcome the chemotherapeutic drug resistance associated with apoptosis deficiency. Besides, the combined application of pyroptosis inducers can enhance the therapeutic effect of traditional chemotherapy drugs. Recent studies also found a close relationship between pyroptosis and antitumor immunity. Next, we will systematically summarize the therapeutic role of pyroptosis in different tumors.

### Lung Cancer

GSDMD is highly expressed in non-small cell cancer (NSCLC) and associated with poor prognosis in lung adenocarcinoma. The knockdown of GSDMD restrains NSCLC cell proliferation *via* intrinsic mitochondrial apoptotic pathways and inhibiting EGFR/Akt signaling (53). Numerous studies have demonstrated that GSDMD played a key role in the proliferation and death of NSCLC cells. Highly expressed GSDMD also improved the possibility to eliminate tumors through the canonical pyroptosis pathway. In recent years, several substances, including 4-hydroxybenacid, Simvastatin, huaier extract, polyphyllin VI, Resibufogenin, have been proved to kill NSCLC *via* caspase-1-dependent pyroptosis (54–58). Polyphyllin VI could elevate ROS/NF-κB signaling to activate canonical pyroptosis in A549 and H1299 cell lines. NAC, a ROS scavenger, could reverse Polyphyllin VI-induced pyroptosis accompanied by suppression of NF-κB, NLRP3, and caspase-1 (57). Interestingly, upregulated ROS restrained NF-κB during pyroptosis induced by Resibufogenin. Thus, the role of NF-κB remains elusive. Additionally, Resibufogenin was found to suppress metastasis by caspase-1 activation, which is manifested as downregulated MMP-2, downregulated MMP-9 and upregulated TIMP-3, further strengthening its antitumor effect (58). P53 was also involved in the regulation of NSCLC canonical pyroptosis. Analysis of tumor samples from NSCLC patients found that P53 was positively correlated with pyroptosis at mRNA and protein levels. Overexpression or silencing of P53 in A549 cells elicited or suppressed pyroptosis respectively. The result from immune immunoprecipitation also verified the interaction between P53 and NLRP3. Furthermore, P53 was significantly upregulated in LPS-induced A549 pyroptosis. In conclusion, P53 might promote pyroptosis *via* binding and activating NLRP3 directly (59). Non-coding RNA also participates in the canonical pyroptosis of NSCLC. Bioinformatics analysis showed that LncRNA-XIST was highly expressed in NSCLC and negatively correlated with the average survival of cancer patients. Thus, inhibiting LncRNA-XIST may contribute to the treatment of NSCLC patients. Subsequent experiments found that knocking down LncRNA-XIST led to pyroptosis by activating the miR-335/SOD2/ROS/NLRP3 signal pathway (60). In another research, downregulation of LncRNA-XIST was demonstrated to relieve its suppression of Smad2 nuclear translocation, subsequently triggering transcriptional activation of P53 and NLRP3. Therefore, the relationship between XIST and pyroptosis was verified from multiple perspectives (61). In addition to NLRP3/caspase-1/GSDMD pathway, the induction of GSDME cleavage in NSCLC causes pyroptosis and inhibits proliferation as well. Diverse molecular targeted agents, including trametinib, erlotinib, ceritinib, elicited both apoptosis and secondary pyroptosis of NSCLC. The pyroptosis was achieved through activation of the caspase-3/GSDME signal pathway mediated by mitochondrial intrinsic apoptosis (62). ROS and NF-κB were also involved in GSDME-dependent pyroptosis. L61H10, a thiopyran derivative, induced an apoptosis-to-pyroptosis switch through inhibiting NF-κB (63). NF-κB suppression was also observed during GSDME-dependent pyroptosis triggered by Piperlongumine analogue L50377. The inhibitory effect on NF-κB was associated with elevated cellular ROS (64). As an inflammatory programmed cell death, pyroptosis also participates in the immunotherapy of NSCLC. In a recent study, researchers designed and constructed a new type of CCCR-modified NK92 cells that overcome the immunosuppressive microenvironment and kill H1299 lung cancer cells through GSDME-dependent pyroptosis. Although the upstream signaling pathway of GSDME activation by NK cells was not well elaborated, the experiment was an interesting attempt to associate pyroptosis with tumor immunity (65). Similar to NK cells, CD8+ T lymphocytes also exhibit the antitumor effect. Compared with unactivated CD8+ T lymphocytes, GSDMD was dramatically upregulated in activated T cells. caspase-4/11 cleaved GSDMD and enhanced cytolytic capacity of the lymphocytes to promoting the killing effect of H1299 cells. Meanwhile, GSDMD-NT was found to co-localize with granzyme B in vesicles which polarized toward the immune synapse of T lymphocytes (**Table 2**). It was speculated that CD8+ T cells might bind to target cells and induce cell death by releasing GSDMD-NT (67).

**Table 2:** Summary of pyroptosis introductive treatment in lung cancer.

Treatment	Cancer Types	Mechanisms of Pyroptosis Induction	Reference
Simvastatin, Huaier extract	NSCLC	NLRP3/Caspase-1/IL-1β & IL-18	(54, 56)
4-hydroxybenzoic acid	NSCLC	Caspase-1/IL-1β & IL-18	(55)
Polyphyllin VI	NSCLC	ROS/NF-κB/NLRP3/Caspase-1/GSDMD/IL-1β & IL-18	(57)
Resibufogenin	NSCLC	ROS/NF-κB suppression/NLRP3/Caspase-1/GSDMD/IL-1β & IL-18	(58)
LPS	NSCLC	P53/NLRP3/Caspase-1	(59)
*LncRNA-XIST* Knock-down	NSCLC	*miR-335*/SOD2/ROS/NLRP3/Caspase-1	(60)
Trametinib, Erlotinib, Ceritinib	NSCLC	BIM/BAX/Cytochrome c/APAF1/Smac/Caspase-9/Caspase-3/GSDME	(62)
Paclitaxel, Cisplatin	NSCLC	Caspase-3/GSDME	(66)
L61H10	NSCLC	NF-κB suppression/GSDME	(63)
L50377	NSCLC	ROS/NF-κB suppression/GSDME	(64)
CCCR-modified NK92 cells	NSCLC	GSDME	(65)

### Digestive System Tumor

In contrast to normal tissues, caspase-1 is low-expressed in hepatocellular carcinoma (HCC). Relevant studies have shown that berberine, euxanthone, alpinumisoflavone and other drugs could activate caspase-1-dependent pyroptosis and kill HCC cells (68–70). Inhibiting autophagy by chloroquine or ATG5 siRNA enhanced alpinumisoflavone induced pyroptosis (70). Meanwhile, 17β-estradiol suppressed autophagy in HCC cells *via* activating NLRP3/caspase-1 (71). Based on these findings, autophagy was hypothesized to play a protective role in drug-induced HCC cell death. In addition to autophagy inhibitors, nanomaterials were also applied to enhance the killing effect of pyroptosis on tumors. A nano-drug delivery system mPEG-PLGA-PLL loaded with arsenic trioxide was designed to improve the therapeutic effect of arsenic trioxide on HCC. The results showed that As2O3 and As2O3-NPs induced pyroptosis differently in diverse cell lines. Caspase-3/GSDME was activated in Huh7 and HepG2 cells, while GSDMD was activated in Bel-7402 cells. Besides, nanoparticles improved the efficacy of As2O3 by enhancing the endocytosis of tumor cells (72). Sorafenib was found to elicited pyroptosis in tumor-associated macrophages (MΦ). Then IL-18 and CCL5 released from pyroptotic MΦ enhanced the chemotaxis and cytotoxicity of NK cells and eliminated HCC *via* activating antitumor immunity. Blockage of IL-1β or IL-18 could terminate Sorafenib’s antitumor effect (73).

The expression levels of caspase-1 and NLRP3 are lower in colorectal cancer (CRC) than adjacent tissues, indicating that pyroptosis associated proteins are negatively correlated with the tumorigenesis and development of CRC. FL118, a camptothecin analog, inhibited proliferation, invasion and metastasis in SW480 and HT129 cells *via* caspase-1-dependent pyroptosis (74). LXRβ was found to interact with pannexin one on the cell membrane, causing the release of ATP into the extracellular environment, further activating P2×7 receptor and NLRP3-dependent pyroptosis (75). c9, t11, c15-CLNA (CLNA1), and t9, t11, c15-CLNA (CLNA2) all belong to conjugated α-linolenic acid isomers, and both of them showed a potent anti-cancer effect through activating canonical and noncanonical pyroptosis respectively (76). LncRNA is also involved in the regulation of CRC pyroptosis. LncRNA RP1−85F18.6 was reported in CRC to promote proliferation and invasion as well as suppress apoptosis and pyroptosis. Knocking down RP1−85F18.6 cleaved GSDMD to trigger pyroptosis. However, the concrete mechanism of GSDMD cleavage was not well elucidated (77). To address the problem of CXCR4+ colorectal cancer stem cells being resistant to multiple chemotherapeutic drugs, the researchers designed a nanostructured toxin T22-DITOX-H6 which contained diphtheria toxin and the CXCR4 ligand T22. The nanostructured toxin induced pyroptosis in colorectal cancer stem cells by binding T22 to CXCR4, specifically manifested by increased NLRP3 and caspase-11 (78). Lobaplatin, a third-generation platinum anti-neoplastic agent, could remarkably elevate the ROS level in CRC cells and phosphorylate JNK. Then activated JNK caused Bax-dependent mitochondrial damage and cytochrome C release, promoting caspase3/9 cleavage and GSDME-dependent pyroptosis (79). Redundant ROS was also observed in the process of CRC cell apoptosis and pyroptosis induced by the combination of Arsenic trioxide and Ascorbic acid (80).

In gastric cancer, chemotherapeutic drugs such as cisplatin and 5-Fu were found to induce GSDME-dependent pyroptosis, which could be transformed into apoptosis *via* GSDME knockout (81, 82). BIX‐01294 is a newly discovered autophagy inducer that, when combined with cisplatin, enhances the sensitivity of gastric cancer to cisplatin by activating autophagy (82). In the study of cisplatin resistance, overexpression LncRNA ADAMTS9-AS2 could sponge miR-223-3p to activate NLRP3 and trigger pyroptosis in cisplatin-resistant cells (83). Chen et al. found that both Betulinic acid and cisplatin could elicit the cleavage of caspase-1 in esophageal cancer cells and tissues, and a combination of the two caused more significant cell death (84). PELP1 is a scaffolding oncogenic protein that is closely related to the progression and prognosis of esophageal cancer. Metformin could induce pyroptosis *via* upregulating miR-497, followed by inhibition of PELP1 to activate the NLRP3/caspase-1/GSDMD pathway (85). Due to the high expression of GSDME in esophageal cancer, some researchers have found that activation of GSDME also led to esophageal cancer pyroptosis. Alpinumisoflavone induced pyroptosis by activating the caspase-3/GSDME pathway. Knocking out of GSDME could convert the pyroptosis into apoptosis (86). PLK1 inhibitor BI2536 could enhance the sensitivity of esophageal squamous cell carcinoma to cisplatin by inhibiting DNA damage repair and inducing pyroptosis. Cisplatin combined with BI2536 triggered esophageal cancer cell death through caspase-3/GSDME pathway, while BI2536 or cisplatin alone did not exhibit morphological changes of pyroptosis (87).

MST1, a vital component of the Hippo pathway, is low expressed in pancreatic cancer. Overexpression of MST1 could increase cellular ROS, activating caspase-1 to elicit pyroptosis. A large number of studies have shown that MST1 played an anti-cancer role in a variety of tumors. Thus, MST1 could be regarded as a potential biomarker and chemotherapy target (88). Gao et al. found that tumor-cell-derived microparticles (TMP) could be used as carriers of anti-cancer drugs to enhance the therapeutic effect. Methotrexate-loaded tumor-cell-derived Microvesicles (MTX–TMPs) perfusion effectively alleviates biliary obstruction caused by extrahepatic cholangiocarcinoma. Further mechanisms studies showed that MTX - TMPs induced pyroptosis of cholangiocarcinoma through the GSDME pathway. Subsequently, the cellular contents released from pyroptosis cells activated macrophages to release inflammatory factors that recruited neutrophils, further enhancing the therapeutic effect of MTX - TMPs perfusion (89).

### Central Nervous System Tumor

Glioblastoma multiforme (GBM) is the most common malignant tumor of the Central Nervous System with a bleak prognosis. Galangin, a natural flavonoid, can induce apoptosis, pyroptosis and protective autophagy in GBM cells. GSDME-NT was significantly elevated in U87MG and U251 cell lines after Galangin treatment. Furthermore, inhibition of autophagy by 3-MA remarkably enhanced apoptosis and pyroptosis triggered by Galangin. Thus, the combined application of autophagy inhibitors and anti-cancer drugs may be helpful for GBM treatment (90). MicroRNAs are also involved in GBM cell pyroptosis. miR-214 can inhibit cell proliferation and migration of U87 and T98G cells through inactivating caspase-1. Immunofluorescence analysis of the brain tissue demonstrated that the GBM showed higher levels of caspase-1, IL-1β, and IL-18 than adjacent tissues, suggesting that caspase-1 and its substrates could promote GBM proliferation and migration (91).

Another central nervous system tumor associated with pyroptosis is neuroblastoma. Dasatinib-induced neuroblastoma SH-Y5Y cell death exhibited the typical characteristics of pyroptosis. It was accomplished through the activation of caspase-3 and cleavage of GSDMD and GSDME. Inhibiting caspase-3 by the specific inhibitor zDEVD notably diminished GSDME-N fragments (**Table 3**). However, the upstream molecules required for GSDMD cleavage in Dasatinib-induced pyroptosis remained unclear (92).

**Table 3:** Summary of pyroptosis introductive treatment in digestive system cancer.

Treatment	Cancer Types	Mechanisms of Pyroptosis Induction	Reference
Berberine	HCC	Caspase-1	(68)
Euxanthone, Alpinumisoflavone, 17β-estradiol	HCC	NLRP3/Caspase-1/IL-1β & IL-18	(69–71)
As2O3	HCC	Caspase-3/GSDME (In Huh7 and HepG2) GSDMD (In Bel-7402)	(72)
FL118	Colorectal cancer	NLRP3/Caspase-1/IL-1β & IL-18	(74)
LXRβ agonists	Colorectal cancer	LXRβ/Pannexin 1/P2×7 receptor/NLRP3/Caspase-1	(75)
c9, t11, c15-CLNA, t9, t11, c15-CLNA	Colorectal cancer	Caspase-1/GSDMD (c9, t11, c15-CLNA) Caspase-4/5 (t9, t11, c15-CLNA)	(76)
*LncRNA RP1−85F18.6* Knock-down	Colorectal cancer	GSDMD	(77)
T22-DITOX-H6	Colorectal cancer	NLRP3 & Caspase-11	(78)
Lobaplatin	Colorectal cancer	ROS/JNK/Bax-mitochondrial apoptotic pathway/Caspase-3/9/GSDME	(79)
Arsenic trioxide + Ascorbic acid	Colorectal cancer	ROS/Caspase-1/IL-1β & IL-18	(80)
5-fluorouracil, cisplatin	Gastric cancer	Caspase-3/GSDME	(81, 82)
*LncRNA ADAMTS9-AS2*	Gastric cancer	LncRNA ADAMTS9-AS2/*miR-223-3p*/NLRP3/Caspase-1/IL-1β & IL-18	(83)
Betulinic acid, Cisplatin	Esophagus cancer	Caspase-1	(84)
Metformin	Esophagus cancer	*miR-497*/PELP1/NLRP3/Caspase-1/GSDMD	(85)
Alpinumisoflavone BI2536 + Cisplatin	Esophagus cancer	Caspase-3/GSDME	(86, 87)
MST1	Pancreatic Cancer	ROS/Caspase-1/IL-1β & IL-18	(88)
MTX–TMPs	Cholangiocarcinoma	GSDME	(89)

### Melanoma

Eukaryotic elongation factor-2 kinase(eEF-2K) is a negative regulator of protein synthesis. In GSDME-overexpressed melanoma cells, doxorubicin induced GSDME-dependent pyroptosis and autophagy accompanied by increasing eEf-2K. Silencing eEF-2K could enhance doxorubicin-induced pyroptosis by decreasing autophagy, suggested that doxorubicin induced protective autophagy by activating eEF-2K in melanoma cells during pyroptosis (93). In another study, iron ions were found to raise the ROS level induced by carbonyl cyanide m-chlorophenyl hydrazone (CCCP), thus triggering oxidation and oligomerization of mitochondrial outer membrane protein Tom20. Subsequently, the Bax/caspase/GSDME pathway was activated, leading to pyroptosis of melanoma cells. The study also found that iron co-treatment increased the sensitivity of melanoma to ROS-inducing drugs such as sulfasalazine, buthionine sulfoximine and induced pyroptosis. Therefore, supplementing iron could be helpful for the clinical treatment of melanoma patients (27). The combination of BRAF inhibitors and MEK inhibitors is recommended by the FDA for the treatment of BRAF V600E/K mutant melanoma. Clinical evidence has shown that BRAFi + MEKi co-treatment could induce T cell infiltration. However, the specific mechanism by which inhibitors induced antitumor immunity is not clear. Erkes et al. found that BRAFi + MEKi combination induced melanoma pyroptosis and release of HMGB1 *via* activating the caspase-3/GSDME pathway. The released HMGB1 activated dendritic cells to promote inflammatory response, eliciting T cell activation and antitumor immunity. For BRAFi + MEKi-resistant melanoma, switching to other chemotherapeutic drugs such as etoposide that induces pyroptosis may be an effective strategy (94).

### Other Tumors

In a study of 108 breast cancer and 23 benign lesions adjacent to breast cancer, expression levels of pyroptosis associated proteins were negatively correlated with the pathological grade and TNM staging of breast cancer. Low expressed caspase-1, GSDMD, and IL-1β contributed to proliferation, invasion, and metastasis of breast cancer (95). Omega-3 Docosahexaenoic acid activated caspase-1/GSDMD/IL-1βpathway to induce pyroptosis in triple-negative breast cancer (96). Wang et al. constructed a bioorthogonal chemical system in which the cancer-imaging probe phenylalanine trifuoroborate (Phe-BF3) specifically identified tumor cells and desilylated and ‘cleaved’ nanoparticle-conjugated gasdermin, promoting the release of GSDMA3 and pyroptosis in breast cancer 4T1 cells. In the tumor-bearing mice model, pyroptosis of less than 15% of tumor cells led to the elimination of the entire 4T1 mammary tumor graft, which was not observed in immunodeficient mice. Further study showed that pyroptosis induced tumor tissue clearance by activating cytotoxic T cells and CD4+ T helper cells in the tumor microenvironment. Besides, due to the low inflammation level within the tumor microenvironment, the immune checkpoint blockade (particularly anti-PD1) had a low response rate in mammary tumor treatment. Combination of NP-GSDMA3, Phe-BF3 and anti-PD1 notably inhibited tumor growth, which indicated that the pyroptosis associated inflammatory response enhanced the antitumor immunity of checkpoint blockade (97). In ovarian cancer, Nobiletin induced increased ROS production and autophagy, promoting cleavage of GSDMD and GSDME (98). 2-(anaphthoyl)ethyltrimethylammonium iodide (α-NETA) induced pyroptosis in ovarian cancer through caspase-4/GSDMD pathway (99). In cervical cancer, tanshinone II A was found to upregulate caspase-3/9 and GSDMD significantly. Inhibiting miRNA145 reversed tanshinone II A induced pyroptosis (100). Sirt1, highly expressed in cervical cancer, is critical for the development of cervical cancer and is associated with poor clinical outcomes. Knocking down Sirt1 activated AIM2 and caspase-1 to elicit pyroptosis. Besides, treating healthy cervical cancer cells with culture media from pyroptotic cells also caused AIM2/caspase-1-dependent pyroptosis. Therefore, pyroptotic cervical cancer cells might release certain substances that triggered pyroptosis in adjacent tissues (101). Induction of pyroptosis was also found in studies of endometrial cancer treatment. Pyroptosis associated proteins were highly expressed in endometrial cancer. Yang et al. found that Hydrogen induced pyroptosis in endometrial cancer cells by activating Ros and Mitosox/NLRP3/Caspase-1/GSDMD signaling pathway (102). BRD4, a member of the bromodomain and extra terminal domain (BET) family, can bind to histone tails to change chromatin structure and influence multiple physiological processes. BRD4 inhibitor JQ1 suppressed proliferation and epithelial-mesenchymal transition of renal cancer *via* triggering pyroptosis (103). Zhang et al. designed and synthesized a series of novel 3’,5’-diprenylated chalcones to develop new chemotherapy drugs. One of these chalcones(C10) was found to induce both apoptosis and GSDME-dependent pyroptosis through activating PKCδ/JNK signing pathway (38). In nasopharyngeal carcinoma, Taxol activated caspase-1/GSDMD pathway to induce pyroptosis. Inhibition of autophagy could improve Taxol’s cytotoxicity (104). Protein kinase R (PKR), a serine-threonine kinase, can be activated by multiple stress signals. Gasticin was found to activate PKR/JNK/NF-κB/caspase-1 signal pathway to triggering nasopharyngeal carcinoma pyroptosis (105). Besides, lobaplatin was also associated with nasopharyngeal carcinoma pyroptosis, albeit by activating the caspase-3/GSDME pathway. Activating cell inhibitor of apoptosis protein-1/2 (cIAP1/2), inhibiting Ripoptosome (RIPK1/Caspase-8/FADD) or ROS could alleviate lobaplatin induced pyroptosis (106). In osteosarcoma, Dioscin inhibited tumor proliferation by inducing G2/M-phase arrest, apoptosis and GSDME-dependent pyroptosis (107). Anthocyanin induced pyroptosis of oral squamous cancer cells through canonical inflammasome pathway (108). Inflammasome components such as NLRP3 and caspase-1 were low expressed in malignant mesothelioma. Doxorubicin and cisplatin could activate the NLRP3/caspase-1 signal pathway to suppress tumor proliferation, and such pyroptosis was further enhanced with the combination of IL-1 receptor antagonist (109). More drugs inducing pyroptosis in tumors from different tissue sources will be developed for clinical treatment with related research progression (**Table 4**).

**Table 4:** Summary of pyroptosis introductive treatment in other tumors.

Treatment	Cancer Types	Mechanisms of Pyroptosis Induction	Reference
Galangin	Glioma	Caspase-3/GSDME	(90)
Dasatinib	Neuroblastoma	Caspase-3/GSDME GSDMD	(92)
Doxoribici, BRAFi + MEKi, Etoposide	Melanoma	Caspase-3/GSDME	(93, 94)
Iron + CCCP	Melanoma	ROS/Tom20/Bax/Caspase-3/GSDME	(27)
DHA	Breast cancer	Caspase-1/GSDMD/IL-1β	(96)
NP-GSDMA3 + Phe-BF3	Breast cancer	GSDMA3	(97)
Nobiletin	Ovarian cancer	ROS/Autophagy/GSDMD & GSDME	(98)
α-NETA	Ovarian cancer	Caspase-4/GSDMD	(99)
Tanshinone II A	Cervical cancer	miR145/GSDMD/IL-1β & IL-18	(100)
SIRT1 knockdown	Cervical cancer	AIM2/Caspase-1	(101)
Hydrogen	Endometrial cancer	ROS and Mitosox/NLRP3/Caspase-1/GSDMD/IL-1β	(102)
JQ1	Renal cancer	NF-κB suppression/NLRP3/Caspase-1/GSDMD	(103)
3’,5’-diprenylated chalcones	Prostate cancer	PKCδ/JNK/Mitochondrial apoptotic pathway/Caspase-3/GSDME	(38)
Taxol	Nasopharyngeal carcinoma	Caspase-1/GSDMD/IL-1	(104)
Casticin	Nasopharyngeal carcinoma	PKR/JNK/NF-κb/Caspase-1/GSDMD	(105)
Lobaplatin	Nasopharyngeal carcinoma	cIAP1/2 suppression & Ripoptosome & ROS/Caspase-3/GSDME	(106)
Dioscin	Osteosarcoma	Caspase-3/GSDME	(107)
Anthocyanin	Oral squamous cell carcinoma	NLRP3/Caspase-1/GSDMD/IL-1β	(108)
Doxorubicin, Cisplatin	Mesothelioma	NLRP3/Caspase-1	(109)

## Research Hotspots in Pyroptosis and Tumor Therapy

### Exploration of the Upstream Pathways That Activate Tumor Pyroptosis

Caspase and gasdermin family are key molecules in pyroptosis. Exploring the upstream pathways that activate pyroptosis associated molecules contributes to an in-depth understanding of pyroptosis in tumors. ROS has been widely involved in tumor pyroptosis induced by various drugs. On the one hand, ROS activates canonical inflammasome pathway to elicit pyroptosis (57, 60). On the other hand, ROS triggers apoptosis and secondary GSDME-dependent pyroptosis (27, 79). Activation of NF-κB is intimately related to cancer development. Thus, regulating NF-κB activity becomes an important strategy for cancer therapy. Resibufogenin, L61H10, JQ1 and other drugs can activate pyroptosis *via* inhibiting NF-κB (58, 63, 103). However, Polyphyllin VI and Casticin were reported to activate NF-κB and caspase-1-dependent pyroptosis (57, 105). Depending on different tumor and drug types, NF-κB showed both oncogenic and anti-cancer effects. As a transcription factor, NF-κB increases transcription of several proto-oncogenes, promoting tumor growth and invasion (103). Meanwhile, NF-κB binds to the NLRP3 promoter, subsequently increasing NLRP3 expression and activating pyroptosis (110). The regulation effect of NF-κB on pyroptosis needs further investigation. JNK, a member of the MAPK family, responds to various stress signals and plays vital roles in regulating apoptosis and pyroptosis (111). Lobaplatin upregulates JNK to enhance mitochondrial translocation of BAX, activating GSDME-dependent pyroptosis *via* mitochondrial apoptosis pathway (79). In recent years, more and more researchers focus their attention on the regulatory role of non-coding RNAs in tumorigenesis and development. Some miRNAs and LncRNAs have been identified to be associated with tumor pyroptosis. Generally, miRNAs regulate cell function by binding to the 3’UTR of target mRNA. Depending on the downstream target, specific miRNAs can act as either oncogenes or tumor suppressor genes. In esophageal cancer, miR-497 inhibited PELP1 to activated caspase-1-dependent pyroptosis (85). In Hela cells, miR-145 upregulated GSDMD, inducing pyroptosis (100). LncRNAs can regulate cell function by targeting miRNAs. Inhibiting LncRNA XIST upregulated miR-335 and induced pyroptosis in NSCLC (60). With the continuous development of related research, plenty of molecules such as Sirt, P53, BIM, etc., have been involved in tumor pyroptosis pathways. The exploration of upstream signal molecules of pyroptosis in tumors will help us further understand the role of pyroptosis in tumors and screen new tumor biomarkers and potential therapeutic targets.

### Develop New Therapy Based on Pyroptosis

#### Screen Effective Chemotherapeutic Drugs

In general, most clinical chemotherapy drugs inhibit tumor growth, invasion and metastasis *via* inducing apoptosis. However, in neurofibroma SH-SY5Y or melanoma MeWo cells with high GSDME expression, traditional chemotherapeutic drugs such as doxorubicin, cisplatin can induce pyroptosis through caspase-3/GSDME pathway (25). As mentioned above, more and more chemotherapeutic drugs have been found to trigger pyroptosis through canonical or noncanonical inflammasome pathway. These studies greatly enriched our understanding of the mechanisms of chemotherapeutic drugs and provided a possibility for future drug discovery and development. Natural compounds such as berberine and casticin can activate pyroptosis to inhibit cancer proliferation (68). The use of compounds that are widely available and easy to extract would help reduce the cost of clinical chemotherapy. Besides, the structure of some natural compounds offers prospective possibilities for the synthesis and development of new chemotherapeutic drugs (38). Actively screening and researching chemotherapeutic drugs, natural compounds and artificially synthesized reagents that induce pyroptosis will further improve the existing treatment for malignant tumors.

#### The Combined Application of Pyroptosis Inductors and Other Anticancer Drugs

Drug combination has become an important strategy for tumor treatment. The combination of chemotherapeutic drugs with independent mechanisms effectively reduces drug resistance and side effects (112). As a newfound PCD, pyroptosis has shown a bright prospect in combined chemotherapy. In esophageal cancer, betulinic acid elicited caspase-1-dependent pyroptosis. Further study demonstrated that the combination of betulinic acid and cisplatin showed stronger growth inhibition than only one alone (84). As a homeostatic degradative process, autophagy is widely involved in regulating tumor cell proliferation and death. Autophagy inducer BIX-01294 was reported to enhance pyroptosis induced by cisplatin, although the specific mechanisms of autophagy activation were unclear (82). In more cases, however, autophagy plays a role in promoting tumor survival. For instance, the combination of autophagy inhibitor and pyroptosis inductors such as doxorubicin, Taxol significantly suppressed tumor proliferation (93, 104).

### Pyroptosis and Antitumor Immunity

As inflammatory cell death, pyroptosis possesses the ability to activate the immune system. Immune stimulants including HMGB1 released from pyroptotic tumor cells can induce the activation of dendritic cells and antitumor T cells. Researchers have found that BRAFi + MEKi treatment in melanoma promoted GSDME-dependent pyroptosis and intensive immune response due to the release of HMGB1 (94). In another study, a biorthogonal system was constructed for selectively releasing active gasdermins into tumor cells. Although the tumor grafts under treatment stopped growth completely, only 10%–30% of the tumor cells underwent pyroptosis. Besides, the tumor regression was abolished in immune-deficient mice (97). All the above results suggested that pyroptosis could play an antitumor role by activating the immune response. On the other hand, tumor-infiltrating immune cells have also been demonstrated to induce pyroptosis of tumor cells. Genetically engineered T cells modified with chimeric antigen receptors (CARs) could induce GSDME-dependent pyroptosis in leukemic cells. What’s more, the activated GSDME in leukemic cells was cleaved by granzyme B released from chimeric antigen receptor (CAR) T cells (113). Granzyme A was also proved to induce tumor cell pyroptosis *via* the cleavage of GSDMB in another recent study (35). The granzyme family seems to play an essential role in pyroptosis triggered by cytotoxic lymphocytes. Furthermore, the activation of pyroptosis pathways in cytotoxic lymphocytes can enhance their cytotoxicity. Researchers have found that GSDMD was upregulated in activated CD8+ T cells and GSDMD deficiency could reduce its cytolytic capacity (67). Immune checkpoint inhibitors (ICIs) have shown a brilliant application prospect in clinical cancer therapy. However, only one-third of patients are responsive to ICIs (114). Recent research showed a synergistic effect between pyroptosis and ICIs. Induction of pyroptosis in target cells sensitized ICI-resistant tumors to checkpoint blockade (97). Due to the cell membrane defect, pyroptotic cells release a large number of cellular contents to induce intense inflammatory responses and massive infiltration of lymphocytes. Markedly increased lymphocyte infiltration further induces caspase-3-dependent and independent tumor cell pyroptosis, forming positive feedback to improve the antitumor effect (89, 94, 97). In conclusion, the bi-directional relationship between pyroptosis and antitumor immunity will be a potential research hotspot.

### Pyroptosis and Nanotechnology

With the progress of material science, nanotechnology has been widely applied in the field of cancer treatment. Nanoparticles can be used as carriers to enhance the antitumor effect of chemotherapeutic drugs. As2O3-NPs enhanced endocytosis of tumor cells to increase the cellular As2O3 content. Then As2O3 exerted antitumor effects *via* inducing pyroptosis (72). In addition, the application of nanoparticles promotes the distribution of pyroptosis inducers in tumor cells rather than adjacent tissues, which will improve the efficacy and reduce side effects. To address the problem of chemotherapy resistance in CXCR4 overexpressed colon cancer, a nanotoxin composed of the catalytic domain of the diphtheria toxin and CXCR4 ligand T22 was designed. The toxin targeted CXCR4+ colon cancer cells specifically and induced pyroptosis (78). In another study, the GSDMA3-NP which could aggregate and release activated GSDMA3 in tumor cells was synthesized for breast cancer treatment (97).

## Conclusions

Pyroptosis is a newfound inflammatory programmed cell death. Membrane perforation, cell swelling and cell rupture are crucial characteristics of pyroptosis. As inflammatory cell death, pyroptosis is closely associated with tumor growth, proliferation and invasion. Pyroptosis associated proteins such as caspase-1, GSDMD were low expressed in multiple cell types. Therefore, inducing pyroptosis in tumor cells has been a new strategy of cancer treatment. In recent years, various chemotherapy drugs, natural compounds and synthetic medicines were found to induce pyroptosis in different types of cancer cells. Signal molecules such as ROS, NF-κB and non-coding RNAs were widely involved in the activation of pyroptosis. In most cases, pyroptosis is triggered through activating caspase-1/GSDMD or caspase-3/GSDME. Whether the rest members of gasdermin family also induce pyroptosis remains unclear. Studies focused on pyroptosis signaling pathways will provide a new possibility for finding tumor biomarkers and further chemotherapeutic drug development. Apoptosis resistance is the common cause of clinical chemotherapy resistance. Induction of other PCD such as necroptosis, pyroptosis could overcome apoptosis resistance. Thus, the combined application of pyroptosis inductors during chemotherapy will be helpful. Besides, the cellular contents released from pyroptotic tumor cells can promote infiltration of inflammatory cells in the tumor microenvironment and improve antitumor immunity. However, the interaction between pyroptosis and antitumor immunity still needs further research. Nanomaterials are also used in the induction of tumor pyroptosis. Nanomaterials can increase the accumulation of pyroptosis inducers in tumor cells and reduce the cytotoxicity to adjacent tissues, possessing high application value. Some nanoparticles can even trigger pyroptosis directly. In conclusion, induction of pyroptosis holds promise as an effective treatment for malignant tumors.

## Author Contributions

LW wrote the manuscript. XQ has put forward many valuable suggestions in the original and revised manuscripts. In addition, he also helped to collect the literature and redraw **Figure 1**. All authors contributed to the article and approved the submitted version.

## Funding

This work was supported by the National Natural Science Foundation of China (No. 81372697, 81701293, 81772669, 81972346), the Changbaishan Scholar Project of Jilin Province (No. 2013026), Scientific Reasearch Foundation of Jilin Province (20190701051GH, 20200201405JC), and the Achievement Transformation Fund of the First Hospital of Jilin University (JDYYZH-1902040).

## Conflict of Interest

The authors declare that the research was conducted in the absence of any commercial or financial relationships that could be construed as a potential conflict of interest.
